# Imatinib-Induced Psoriasis

**DOI:** 10.4274/Tjh.2012.0147

**Published:** 2013-06-05

**Authors:** Figen Atalay, Ebru Kızılkılıç, R. Simin Ada

**Affiliations:** 1 Başkent University School of Medicine, Department of Hematology, İstanbul, Turkey; 2 Maltepe University, School of Medicine, Department of Hematology, Istanbul, Turkey; 3 Başkent University School of Medicine, Department of Dermatology, İstanbul, Turkey

**Keywords:** Chronic myelogenous leukemia, Imatinib, Psoriasis

## LETTER TO THE EDITOR

Imatinib is a signal transduction regulator that selectively inhibits the tyrosine kinase family, including bcr-abl and c-kit, and the platelet-derived growth factor (PDGF) receptor. It is currently the first-line therapy for newly diagnosed chronic myeloid leukemia (CML) patients [1]. We report the case of a patient who had no previous history of psoriasis but developed psoriasis after starting imatinib.

**Figure 1:**
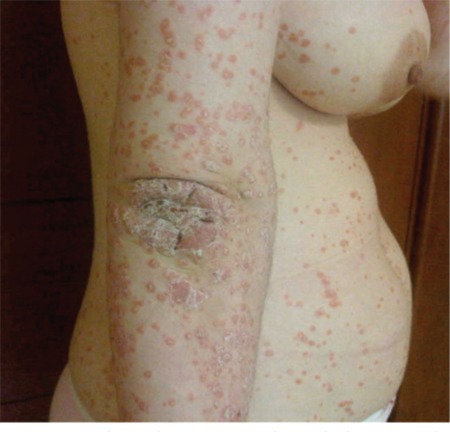
Scaly erythematous papules and plaques on the right arm

**Figure 2:**
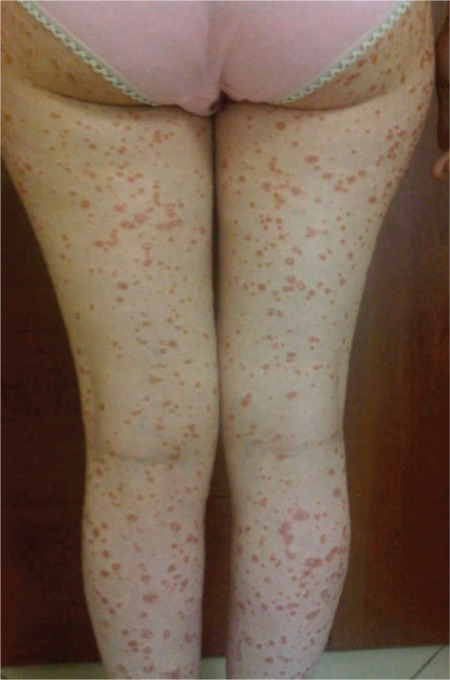
Erythematous papulosquamous lesions on the lower extremities

**Figure 3:**
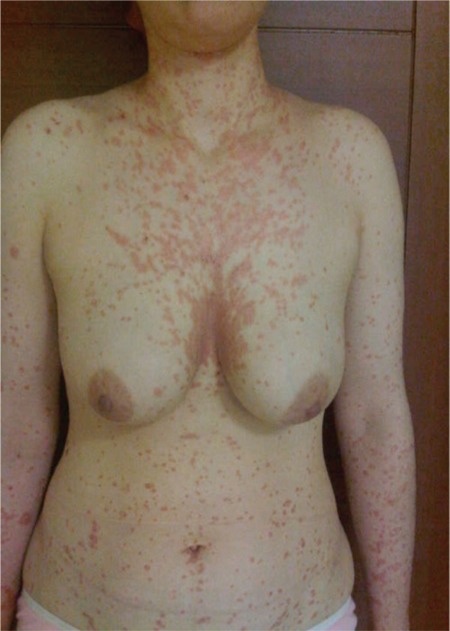
Scaly erythematous papules and plaques on the neck, upper extremities, and and trunk

**Figure 4:**
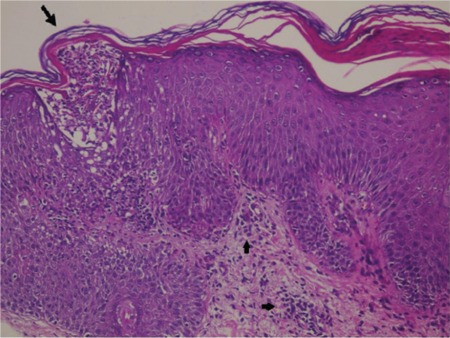
Parakeratosis, leukocyte abscesses in the keratin layer (big arrow), psoriasiform hyperplasia of the epithelium, loss of the granular layer areas of parakeratosis, leukocyte abscesses in the surface epithelium, superficial perivascular lymphocytes (small arrows), extravasated erythrocytes.

A 21-year-old woman was diagnosed with CML in the chronic phase. Imatinib mesylate was started at a daily dose of 400 mg. The patient achieved a complete hematological response within 3 months. Five months after her CML diagnosis and imatinib usage, she developed an erythematous scaly eruption with plaques of various sizes on her trunk and extremities (Figures 1, 2, and 3). She had no previous history of psoriasis and had not taken any drugs except for imatinib, nor did she have any relatives with a history of psoriasis. The patient underwent a skin biopsy, which revealed a neutrophilic scale crust and loss of the granular cell layer, which are most consistent with psoriasis (Figure 4). The discontinuation of imatinib treatment and subsequent introduction of narrow-band ultraviolet B therapy improved the skin condition, and her psoriatic skin lesions had almost disappeared within 3 weeks. Since that time, nilotinib has been started. So far, the patient has not complained of any cutaneous side effects, and she achieved a complete cytogenetic response at 6 months and remains clinically well, currently receiving nilotinib at a dose of 200 mg twice daily.

Cutaneous reactions to imatinib are common and may occur in 7% to 88.9% of patients in different series. Maculopapular eruptions, erythematous eruptions, edema, and periorbital edema are the most common adverse events seen [2]. In 2002, Miyagawa et al. reported a patient who had intractable psoriasis but experienced significant improvement while being treated with imatinib for concomitant metastatic gastrointestinal stromal tumors [3]. Valeyrie et al. also reported psoriatic dermatological changes in 4 out of 54 patients who were using imatinib. Two of these 4 patients had no history of psoriasis [2,4]. Psoriasis has long been identified as an immune disorder in which T lymphocytes play a primary role in the pathogenesis. Imatinib affects cytokine production and the proliferation of T cells and inhibits the secretion of interferon-c by T effector cells. These effects, together with imatinib’s suppression of c-kit and PDGF receptors, may help to explain the exacerbation of psoriasis in some patients [5,6,7,8,9]. The cause of imatinib-related nonpsoriatic forms of skin lesions is not clear. The most probable cause is the fact that imatinib affects mast cells. Because mast cells express a functional c-kit, which is susceptible to imatinib, this drug causes mast cells to proliferate. Another mechanism involves chemoattractant substances, such as cytokines and growth factors, which can lead to an accumulation of dermal mast cells. Imatinib-related skin toxicities are usually dose-dependent, and skin biopsies have shown a mixed cellular infiltrate [10]. In our case, we replaced imatinib with nilotinib therapy, and the psoriasis has not recurred over the course of about 1 year. The patient still maintains a complete molecular and hematological response. Imatinib-induced skin reactions can be self-limiting, but occasionally drug withdrawal is required. This case demonstrates that imatinib can cause psoriasis to occur or can exacerbate the condition. In cases where patients using imatinib develop psoriasis, nilotinib can be a safe alternative.
